# Differential regulation of Aβ42-induced neuronal C1q synthesis and microglial activation

**DOI:** 10.1186/1742-2094-2-1

**Published:** 2005-01-10

**Authors:** Rong Fan, Andrea J Tenner

**Affiliations:** 1 Department of Molecular Biology and Biochemistry, Institute of Brain Aging and Dementia, University of California, Irvine, Irvine, CA 92697 USA

## Abstract

Expression of C1q, an early component of the classical complement pathway, has been shown to be induced in neurons in hippocampal slices, following accumulation of exogenous Aβ42. Microglial activation was also detected by surface marker expression and cytokine production. To determine whether C1q induction was correlated with intraneuronal Aβ and/or microglial activation, D-(-)-2-amino-5-phosphonovaleric acid (APV, an NMDA receptor antagonist) and glycine-arginine-glycine-aspartic acid-serine-proline peptide (RGD, an integrin receptor antagonist), which blocks and enhances Aβ42 uptake, respectively, were assessed for their effect on neuronal C1q synthesis and microglial activation. APV inhibited, and RGD enhanced, microglial activation and neuronal C1q expression. However, addition of Aβ10–20 to slice cultures significantly reduced Aβ42 uptake and microglial activation, but did not alter the Aβ42-induced neuronal C1q expression. Furthermore, Aβ10–20 alone triggered C1q production in neurons, demonstrating that neither neuronal Aβ42 accumulation, nor microglial activation is required for neuronal C1q upregulation. These data are compatible with the hypothesis that multiple receptors are involved in Aβ injury and signaling in neurons. Some lead to neuronal C1q induction, whereas other(s) lead to intraneuronal accumulation of Aβ and/or stimulation of microglia.

## Introduction

Alzheimer's disease (AD) is the most common form of dementia in the elderly. Its main pathological features include extracellular amyloid beta (Aβ) deposition in plaques, neurofibrillary tangles (composed of hyperphosphorylated tau protein) in neurons, progressive loss of synapses and cortical/hippocampal neurons, and upregulation of inflammatory components including activated microglia and astrocytes and complement activation [1]. Although the contribution of abnormal phosphorylation and assembly of tau to AD dementia remains a focus of investigation, therapies that interfere with Aβ production, enhance its degradation, or cause its clearance from the central nervous system (CNS) have been the center of many studies in search of a cure for this disease.

Microglial cells, when activated, are believed to be responsible for much of the Aβ clearance through receptor-mediated phagocytosis [2,3]. Upon activation, microglia acquire features more characteristic of macrophages, including high phagocytic activity, increased expression of leukocyte common antigen (CD45), major histocompatibility complex (MHC) class II and costimulatory molecules B7, and secretion of proinflammatory substances [4]. In addition, phagocytic microglia also participate in the removal of degenerating neurons and synapses as well as Aβ deposits ([5], and reviewed in [6]). Thus, while some microglial functions are beneficial, the destructive effects of the production of toxins (such as nitric oxide, superoxide) and proinflammatory cytokines by activated microglia apparently overcome the protective functions in the chronic stage of neuroinflammation [7,8]. *In vitro *studies have shown both protection and toxicity contributed by microglia in response to Aβ depending on the state of activation of microglia [9,10]. Correlative studies on AD patients and animal models of AD strongly suggest that accumulation of reactive microglia at sites of Aβ deposition contributes significantly to neuronal degeneration [3,11], although decreased microglia have been reported to be associated with both lowered and enhanced neurodegeneration in transgenic animals [12,13]. Aβ itself is believed to initiate the accumulation and activation of microglia. However, recent reports provide evidence for neuron-microglial interactions in regulating CNS inflammation [14]. Nevertheless, the molecular mechanisms responsible for activation and regulation of microglia remain to be defined.

Complement proteins have been shown to be associated with Aβ plaques in AD brains, specifically those plaques containing the fibrillar form of the Aβ peptide [11]. Complement proteins are elevated in neurodegenerative diseases like AD, Parkinson's disease, and Huntington's disease as well as more restricted degenerative diseases such macular degeneration and prion disease [11,15-18]. Microglia, astrocytes, and neurons in the CNS can produce most of the complement proteins upon stimulation. C1q, a subcomponent of C1, can directly bind to fibrillar Aβ and activate complement pathways [19], contributing to CNS inflammation [13]. In addition, C1q has been reported to be synthesized by neurons in several neurodegenerative diseases and animal injury models, generally as an early response to injury [20-23], possibly prior to the synthesis of other complement components.

Interestingly, C1q and, upon complement activation, C3 also can bind to apoptotic cells and blebs and promote ingestion of those dying cells [24-26]. Elevated levels of apoptotic markers are present in AD brain tissue suggesting that many neurons undergo apoptosis in AD [27-29]. Excess glutamate, an excitatory neurotransmitter released from injured neurons and synapses, is one of the major factors that perturb calcium homeostasis and induce apoptosis in neurons [30]. Thus, it is reasonable to hypothesize that neuronal expression of C1q, as an early injury response, may serve a potentially beneficial role of facilitating the removal of apoptotic neurons or neuronal blebs [31] in diseases thereby preventing excess glutamate release, excitotoxicity, and the subsequent additional apoptosis.

We have previously reported that in rat hippocampal slice cultures treated with exogenous Aβ42, C1q expression was detected in pyramidal neurons following the internalization of Aβ peptide. This upregulation of neuronal C1q could be a response to injury from Aβ that would facilitate removal of dying cells. Concurrently, microglial activation was prominent upon Aβ treatment. In the present study, the relationship of Aβ-induced neuronal C1q production to microglia activation and Aβ uptake in slice cultures was investigated.

## Materials and methods

### Materials

Aβ 1–42, obtained from Dr. C. Glabe (UC, Irvine), was synthesized as previously described [32]. Aβ 10–20 was purchased from California Peptide Research (Napa, CA). Lyophilized (in 10 mM HCl) Aβ peptides were solubilized in H_2_O and subsequently N-2-hydroxyethylpiperazine-N'-2-ethanesulfonic acid (HEPES) was added to make a final concentration of 10 mM HEPES, 500 μM peptide. This solution was immediately diluted in serum-free medium and added to slices. Glycine-arginine-glycine-aspartic acid-serine-proline (RGD) peptide was purchased from Calbiochem (San Diego, CA). D-(-)-2-amino-5-phosphonovaleric acid (APV) was purchased from Sigma (St. Louis, MO). Both compounds were dissolved in sterile Hanks' balanced salt solution (HBSS) without glucose at 0.2 M and 5 mM, respectively, before diluted in serum-free medium. Antibodies used in experiments are listed in Table 1; RT-PCR primers, synthesized by Integrated DNA Technologies (Coralville, IA), are listed in Table 2. All other reagents were from Sigma unless otherwise noted.

**Table 1:** Antibodies used in immunohistochemistry.

**antibody/antigen**	**concentration**	**source**
anti-rat C1q	2 μg/ml	M. Wing, Cambridge, UK
OX-42 (CD11b/c)	5 μg/ml	BD/PharMingen, San Diego, CA
ED-1	3 μg/ml	Chemicon, Temecula, CA
anti-CD45	0.5 μg/ml	Serotec Inc, Raleigh, NC
4G8 (Aβ)	1 μg/ml	Signet Pathology Systems, Dedham, MA
6E10 (Aβ)	0.5 μg/ml	Signet Pathology Systems

**Table 2:** PCR primers and cycling conditions for RT-PCR assay.

Gene	Primer sequences	Denaturation	Annealing	Extension	cycle	Ref
C1qB	5'-cgactatgcccaaaacacct-3' 5'-ggaaaagcagaaagccagtg-3'	94°C 1 min	60°C 1 min30 sec	72°C 2 min	35	[61]
MCSF	5'-ccgttgacagaggtgaacc-3' 5'-tccacttgtagaacaggaggc-3'	92°C 30 sec	58°C 1 min	72°C 1 min30 sec	35	[62]
CD40	5'-cgctatggggctgcttgttgacag-3' 5'-gacggtatcagtggtctcagtggc-3'	94°C 30 sec	58°C30 sec	72°C 1 min	30	[63]
β-actin	5'-ggaaatcgtgcgtgacatta-3' 5'-gatagagccaccaatccaca-3'	94°C 30 sec	60°C30 sec	72°C 1 min	25	[61]
IL-8	5'-gactgttgtggcccgtgag-3' 5'-ccgtcaagctctggatgttct-3'	94°C 1 min	56°C 1 min	72°C 1 min	39	[64]

### Slice cultures

Hippocampal slice cultures were prepared according to the method of Stoppini et al [33] and as described in Fan and Tenner [34]. All experimental procedures were carried out under protocols approved by the University of California Irvine Institutional Animal Care and Use Committee. Slices prepared from hippocampi dissected from 10d-old Sprague Dawley rat pups (Charles River Laboratories, Inc., Wilmington, MA) were kept in culture for 10 to 11 days before treatment started. All reagents were added to serum-free medium (with 100 mg/L transferrin and 500 mg/L heat-treated bovine serum albumin) which was equilibrated at 37°C, 5% CO_2 _before addition to the slices. Aβ 1–42 or Aβ 10–20 was added to slice cultures as described previously [34]. Briefly, peptide was added to cultures in serum-free medium at 10 or 30 μM. After 7 hours, the peptide was diluted with the addition of an equal amount of medium containing 20% heat-inactivated horse serum. Fresh peptide was applied for each day of treatment. Controls were treated the same way except without peptide. RGD or APV was added to the slice cultures at the same time as Aβ 42.

### Immunohistochemistry

At the end of the treatment period, media was removed, the slices were washed with serum-free media and subjected to trypsinization as previously described [34] for 15 minutes at 4°C to remove cell surface associated, but not internalized, Aβ. After washing, slices were fixed and cut into 20 μm sections for immunohistochemistry or extracted for protein or RNA analysis as described in Fan and Tenner [34]. Primary antibodies (anti-Aβ antibody 4G8 or 6E10; rabbit anti rat C1q antibody; CD45 (leukocyte common antigen, microglia), OX42 (CD11b/c, microglia), or ED1 (rat microglia/macrophage marker), or their corresponding control IgGs were applied at concentrations listed in Table 1, followed by biotinylated secondary antibody (Vector Labs, Burlingame, CA) and finally FITC- or Cy3-conjugated streptavidin (Jackson ImmunoResearch Laboratories, West Grove, PA). Slides were examined on an Axiovert 200 inverted microscope (Carl Zeiss Light Microscopy, Göttingen, Germany) with AxioCam (Zeiss) digital camera controlled by AxioVision program (Zeiss). Images (of the entire CA1-CA2 region of hippocampus) were analyzed with KS 300 analysis program (Zeiss) to obtain the percentage area occupied by positive immunostaining in a given field.

### ELISA

Slices were homogenized in ice-cold extraction buffer (10 mM triethanolamine, pH 7.4, 1 mM CaCl_2_, 1 mM MgCl_2_, 0.15 M NaCl, 0.3% NP-40) containing protease inhibitors pepstatin (2 μg/ml), leupeptin (10 μg/ml), aprotinin (10 μg/ml), and PMSF (1 mM). Protein concentration was determined by BCA assay (Pierce, Rockford, IL) using BSA provided for the standard curve.

An ELISA for rat C1q was adapted from Tenner and Volkin [35] with some modifications as previously described [34].

### RNA preparation and RT-PCR

Total RNA from cultures was isolated using the Trizol reagent (Life Technologies, Grand Island, NY) according to the manufacturer's instructions. RNA was treated with RNase-free DNase (Fisher, Pittsburgh, PA) to remove genomic DNA contamination. Each RNA sample was extracted from 3 to 5 hippocampal organotypic slices in the same culture insert. The reverse transcription (RT) reaction conditions were 42°C for 50 min, 70°C for 15 min. Tubes were then centrifuged briefly and held at 4°C. Primer sequences and PCR conditions are listed in Table 2. PCR products were electrophoresed in 2% agarose gel in TAE buffer and visualized with ethidium bromide luminescence. To test for differences in total RNA concentration among samples, mRNA level for rat β-actin were also determined by RT-PCR. Results were quantified using NIH image software [36] by measuring DNA band intensity from digital images taken on GelDoc (BIO-RAD) with Quantity One program.

## Results

### NMDA receptor antagonist APV inhibits Aβ42 uptake and Aβ42-induced microglial activation and neuronal C1q production

**Figure 1:**
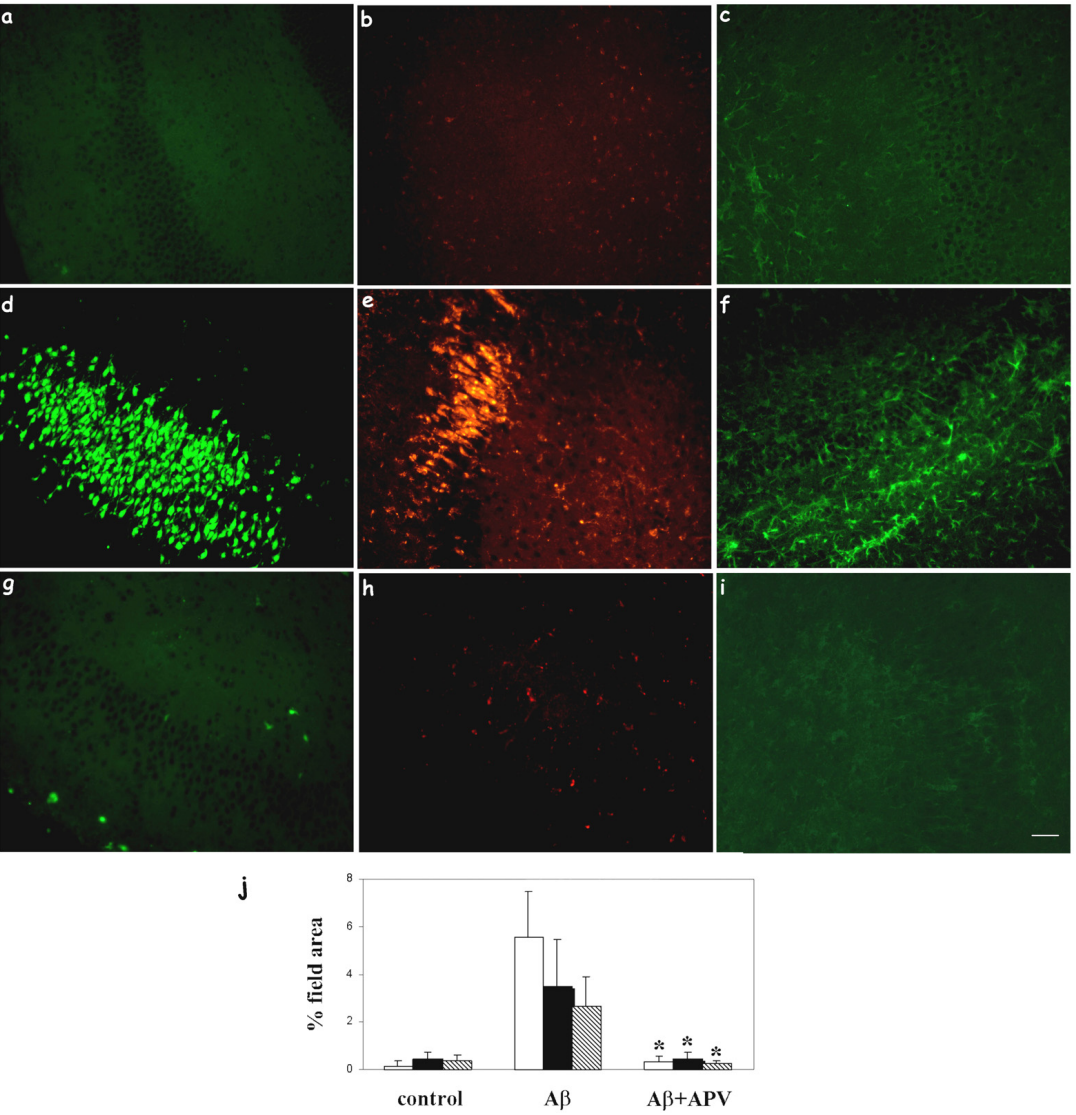
APV inhibited Aβ uptake, neuronal C1q production, and microglial activation. Slices were treated with no peptide (a, b, c), 30 μM Aβ 42 (d, e, f), or 30 μM Aβ 42 + 50 μM APV (g, h, i) for 3 days with fresh reagents added daily. Immunohistochemistry for Aβ (4G8, a, d, g), C1q (anti-rat C1q, b, e, h), and microglia (CD45, c, f, i) was performed on fixed and sectioned slices. Scale bar = 50 μm. Results are representative of three separately performed experiments. j. Immunoreactivity of Aβ (open bar), C1q (black bar), or CD45 (striped bar) was quantified as described in Materials and Methods. Values are the mean ± SD (error bars) from images taken from 8 slices (2 sections per slice) in 3 independent experiments (* p < 0.0001 compared to Aβ, Anova single factor test).

**Figure 2:**
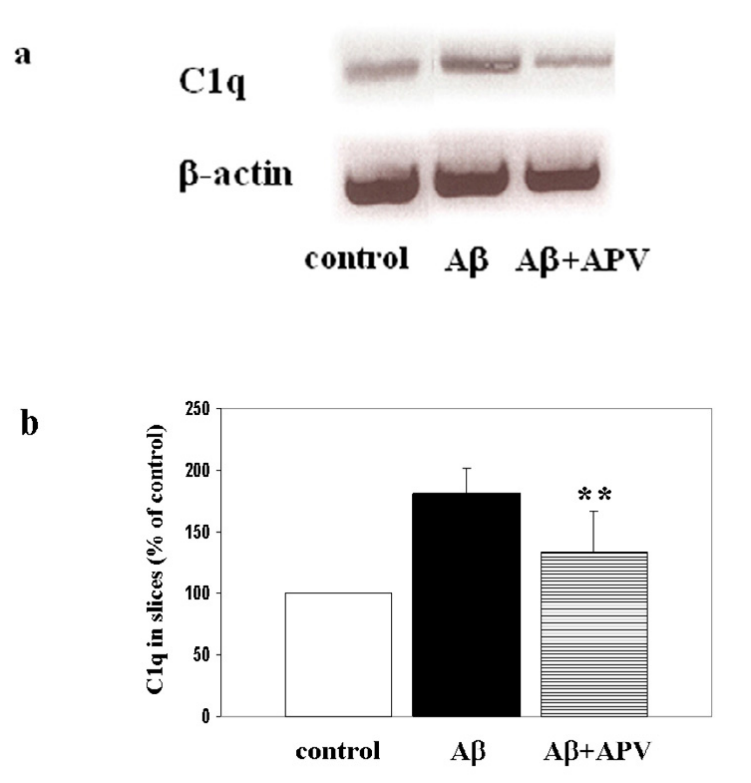
Inhibition of Aβ-induced C1q synthesis by APV. a. C1q and β-actin mRNAs were assessed by RT-PCR in slices after 3 days of no peptide, 30 μM Aβ, or 30 μM Aβ + 50 μM APV treatment. Results are from one experiment representative of two independent experiments. b. Slices were treated with no peptide (open bar), 30 μM Aβ (black bar), or 30 μM Aβ + 50 μM APV (striped bar) daily for 3 days. 3 or 4 slices that had received same treatment were pooled, extracted and proteins analyzed by ELISA. Data are presented as percentage of control in ng C1q/mg total protein (mean ± SD of three independent experiments, **p = 0.01 compared to Aβ, one-tailed paired t-test).

We have previously reported that C1q was detected in cells positive for neuronal markers and that microglial cells were activated in slices following Aβ42 ingestion [34]. Lynch and colleagues have shown that APV, a specific NMDA glutamate receptor antagonist, was able to block Aβ42 uptake by hippocampal neurons in slice cultures [37]. This provided a mechanism to down-modulate the Aβ42 internalization and test the effect on induction of C1q synthesis in neurons. Slices were treated with no peptide, 50 μM APV, 30 μM Aβ42, or 30 μM Aβ42 + 50 μM APV for 3 days with fresh reagents added daily. Cultures were collected and processed as described in Materials and Methods. Similar to reported previously, addition of exogenous Aβ42 resulted in Aβ uptake by hippocampal neurons, induction of C1q synthesis in neurons, and activation of microglial cells (Figure 1d, e, f compared with 1a, b, c). As anticipated, Aβ42 uptake in neurons detected by both 4G8 (Figure 1g) and 6E10 (data not shown) was inhibited by APV co-treatment. Neuronal C1q immunoreactivity was also inhibited when APV was added to Aβ42 treated slices (Figure 1h). Aβ42-triggered microglial activation, assessed by upregulation of antigens detected by anti-CD45 (Figure 1i vs. 1f), OX42 and ED1 (data not shown) was also fully diminished by APV. To quantify the immunohistochemistry results, images were taken from the entire CA1-CA2 region of each immunostained hippocampal section and averaged. Image analysis further substantiated the reduction in Aβ uptake, C1q synthesis and microglial activation (Figure 1j). C1q gene expression at mRNA and protein levels was also assessed by RT-PCR and ELISA, respectively. Results showed decrease of C1q mRNA and protein in slice extracts treated with 30 μM Aβ42 + APV, compared to 30 μM Aβ42 alone (Figure 2a and 2b, n = 2).

### Integrin receptor antagonist GRGDSP (RGD) peptide enhances Aβ42 uptake and Aβ42-induced microglial activation and neuronal C1q expression

It has been shown that an integrin receptor antagonist peptide, GRGDSP (RGD), can enhance Aβ ingestion by neurons in hippocampal slice cultures [37]. Therefore, we adopted this experimental manipulation as an alternative approach to modulate the level of Aβ uptake in neurons and assess the correlation between Aβ ingestion and neuronal C1q expression. Slices were treated with no peptide, 2 mM RGD, 10 μM Aβ42, or 10 μM Aβ42 + 2 mM RGD for 3 days with fresh peptides added daily. At the end of treatments, slices were collected and processed.

**Figure 3:**
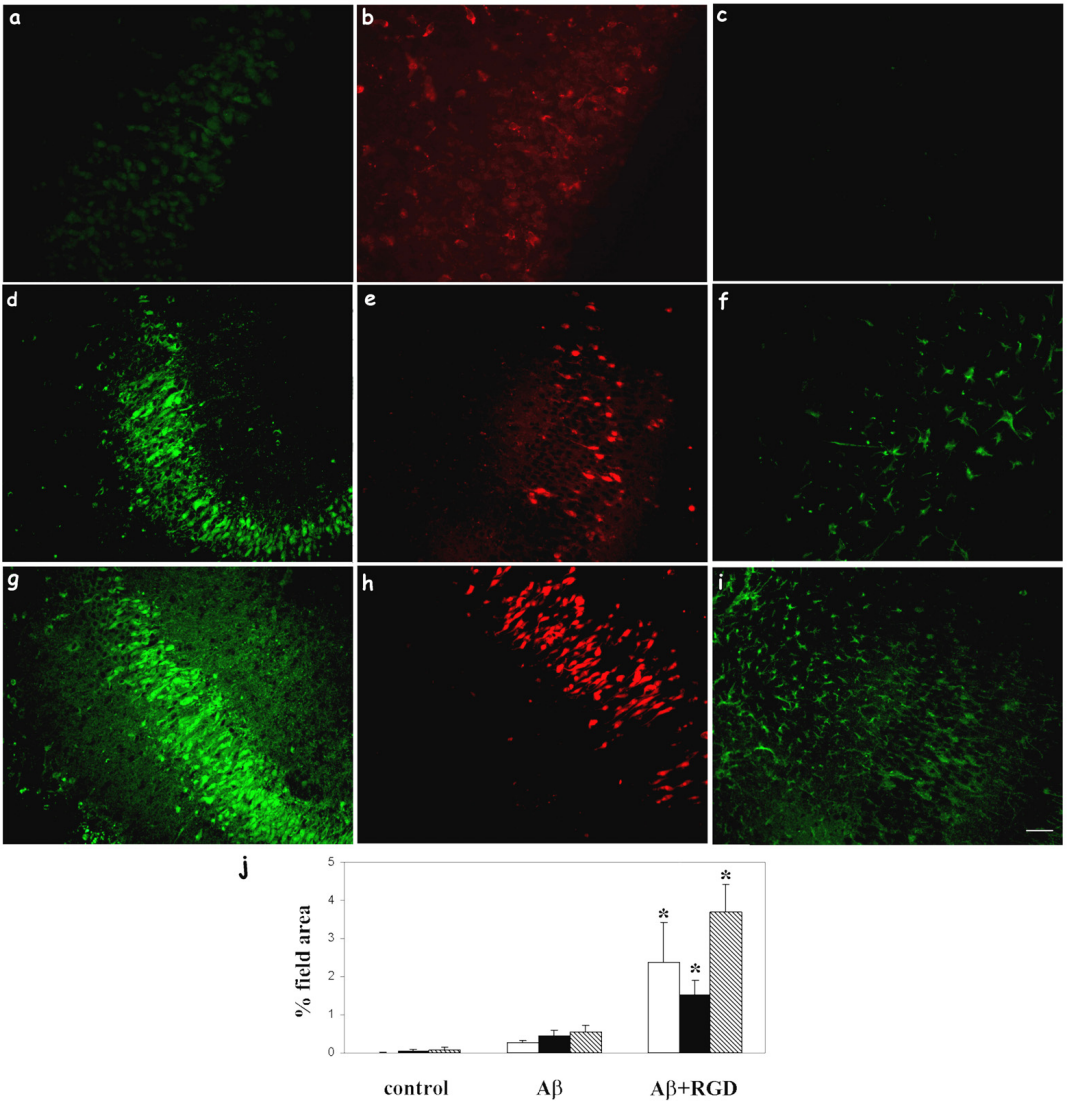
RGD enhanced Aβ uptake, neuronal C1q expression, and microglial activation. Hippocampal slices were treated with no peptide (a, b, c), 10 μM Aβ 42 (d, e, f), or 10 μM Aβ 42 + 2 mM RGD (g, h, i) for 3 days with fresh peptides added daily. Immunohistochemistry for Aβ (4G8, a, d, g), C1q (anti-rat C1q, b, e, h), and microglia (CD45, c, f, i) was performed on fixed slice sections. Scale bar = 50 μm. Results are representative of three separately performed experiments. j. Immunoreactivities of Aβ (open bar), C1q (black bar), or CD45 (striped bar) were quantified as described in Materials and Methods. Values are the mean ± SD (error bars) from images taken from 8 slices (2 sections per slice) in 3 independent experiments (* p < 0.0001, compared to Aβ, Anova single factor test).

**Figure 4:**
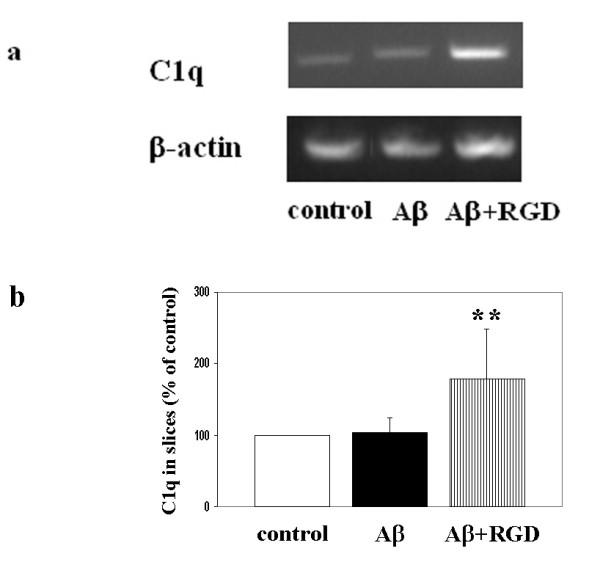
Enhancement of Aβ-induced C1q synthesis by RGD. a. C1q and β-actin mRNAs were assessed by RT-PCR in slices after 3 days of no peptide, 10 μM Aβ, or 10 μM Aβ + 2 mM RGD treatment. Results are from one experiment representative of two independent experiments. b. Slices were treated with no peptide (open bar), 10 μM Aβ (black bar), or 10 μM Aβ + 2 mM RGD (striped bar) daily for 3 days. 3 or 4 slices that had received same treatment were pooled, extracted and proteins analyzed by ELISA. Data are presented as percentage of control in ng C1q/mg total protein (mean ± SD of three independent experiments, **p = 0.06 compared to Aβ, one-tailed paired t-test).

Addition of RGD peptide by itself did not result in neuronal C1q induction or microglial activation (CD45) compared to no treatment control, as assessed by immunostaining (data not shown). While greater ingestion was seen at 30 μM (Figure 1d, e, f), addition of 10 μM Aβ shows detectable Aβ ingestion, C1q expression, and microglial activation (Figure 3d, e, f compared with 3a, b, c). The lower concentration of Aβ was chosen for these experiments to ensure the detection of potentiation of uptake (vs. a saturation of uptake at higher Aβ42 concentrations). When RGD was provided in addition to 10 μM Aβ42, Aβ immunoreactivity in neurons with antibody 4G8 (Figure 3g vs. 3d) and 6E10 (similar results, data not shown), neuronal C1q expression (Figure 3h vs. 3e), and CD45 (Figure 3i vs. 3f) upregulation in microglia triggered by Aβ42, were significantly enhanced. Enhanced microglial activation was also detected with OX42 and ED1 antibodies (data not shown). Quantification by image analysis (Figure 3j) definitively demonstrated that the increased accumulation of Aβ in neurons, microglial activation, and induction of neuronal C1q synthesis in the presence of RGD. RT-PCR (Figure 4a) and ELISA (Figure 4b) further demonstrated that both mRNA and protein expression of C1q was enhanced by RGD. Thus, under the conditions tested, both neuronal C1q synthesis and microglial activation are coordinately affected when the internalization of Aβ is modulated negatively by APV or positively by RGD.

### Aβ10–20 blocks Aβ42 induced microglial activation but triggers C1q synthesis in hippocampal neurons

**Figure 5:**
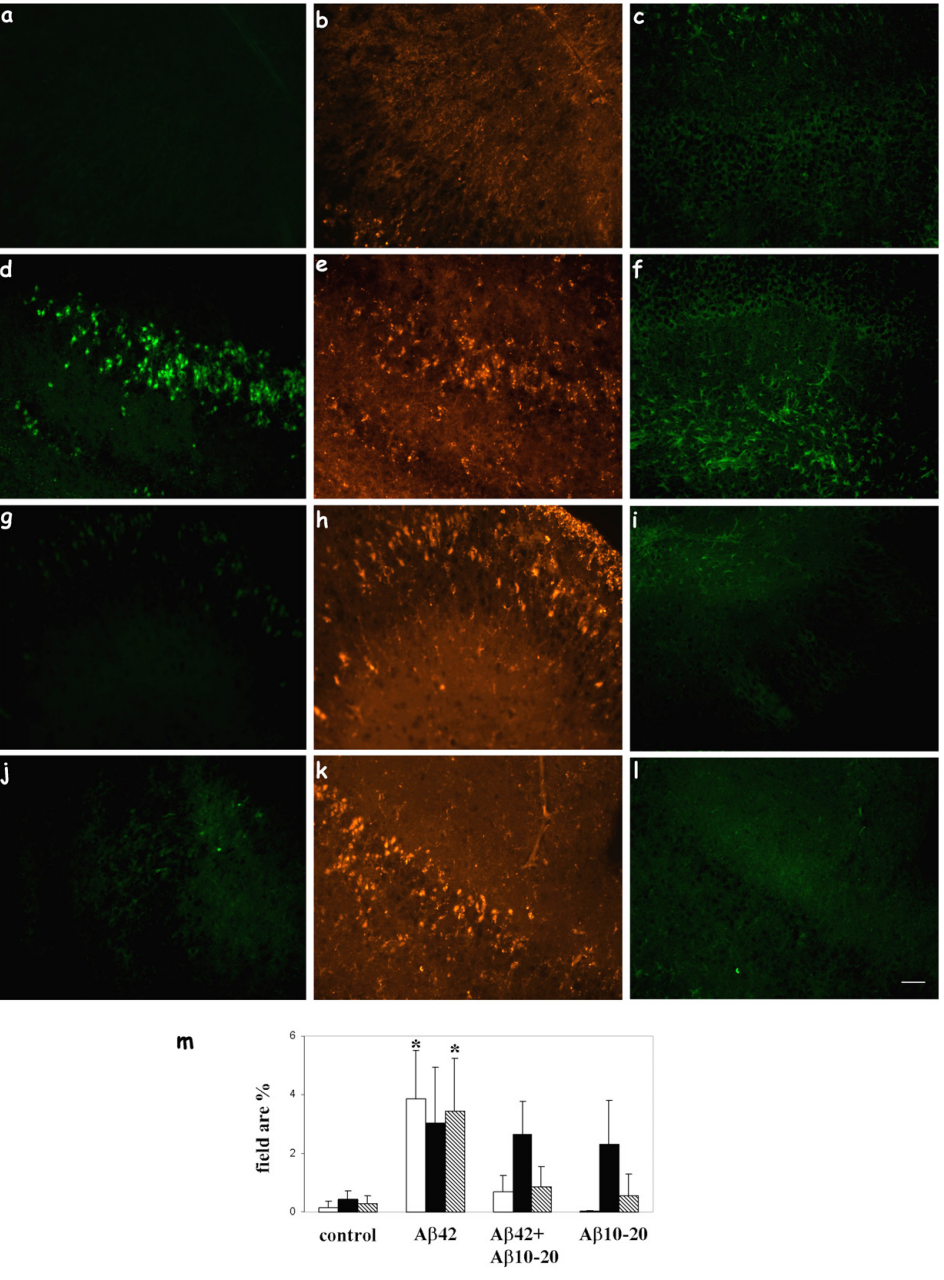
Aβ10–20 blocked Aβ42 uptake, microglial activation, but not neuronal C1q induction. Slices were treated with no peptide (a, b, c), 10 μM Aβ 42 (d, e, f), 10 μM Aβ 42 + 30 μM Aβ 10–20 (g, h, i) or 30 μM Aβ 10–20 (j, k, l) for 3 days with fresh peptides added daily. Immunohistochemistry for Aβ (4G8, a, d, g, j), C1q (anti-rat C1q, b, e, h, k), and microglia (CD45, c, f, i, l) was performed on fixed and sectioned slices. Results are representative of three independent experiments. Scale bar = 50 μm. m. Immunoreactivities of Aβ (open bar), C1q (black bar), or CD45 (striped bar) were quantified as described in Materials and Methods. Values are the mean ± SD (error bars) from images taken from 8 slices (2 sections per slice) in 3 independent experiments. Microglial activation by Aβ42 was significantly inhibited by Aβ10–20 (* p < 0.0001, compared to either Aβ42 + Aβ10–20 or Aβ10–20, Anova single factor test).

**Figure 6:**
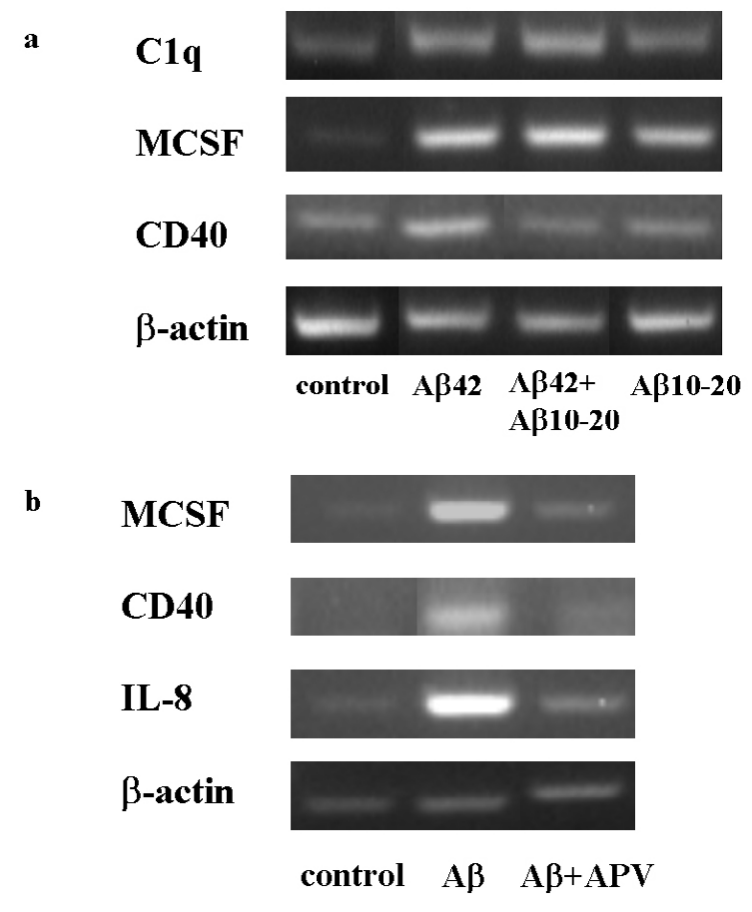
a. Aβ10–20 inhibited Aβ42-induced C1q and CD40 mRNA elevation, but not that of MCSF. C1q, MCSF, CD40, and β-actin mRNAs were assessed by RT-PCR in slices treated for 3 days with no peptide, 10 μM Aβ 42, 30 μM Aβ 10–20, or 10 μM Aβ 42 + 30 μM Aβ 10–20. Results are from one experiment representative of two independent experiments. b. APV blocked MCSF, CD40, and IL-8 mRNA induction triggered by Aβ42. RT-PCR for MCSF, CD40, IL-8, and β-actin were performed on RNA extracted from slices treated with no peptide (control), 30 μM Aβ 42, or 30 μM Aβ42 + 50 μM APV for 3 days. Results are from one experiment representative of two separate experiments.

Data reported by Giulian et al suggests that residues 13–16, the HHQK domain in human Aβ peptide, mediate Aβ-microglia interaction [38]. To investigate the effect of HHQK peptides in this slice culture system, rat hippocampal slices were treated with no peptide, 10 μM Aβ42, 10 μM Aβ42 + 30 μM Aβ10–20, or 30 μM Aβ10–20 for 3 days with fresh peptides added daily. Sections were immunostained for Aβ, C1q, and microglia. Aβ immunoreactivity was significantly reduced in the Aβ42 +Aβ10–20 treated tissues compared to the Aβ42 alone treatment (Figure 5g vs. 5d). Aβ10–20 alone-treated slices lacked detectable immunopositive cells with either 4G8 or 6E10 anti-Aβ antibody (Figure 5j and data not shown). Furthermore, as anticipated [38], when Aβ10–20 was present, microglial activation by Aβ42 as assessed by level of CD45, OX42, and ED1, was significantly reduced (Figure 5i vs. 5f and data not shown). Image analysis confirmed the inhibition of Aβ uptake (Figure 5m, open bars) and microglial activation (Figure 5m, striped bars) by the HHQK-containing Aβ10–20 peptide. However, production of C1q in neurons treated with Aβ42 was not inhibited by Aβ10–20 (Figure 5h vs. 5e). In fact, with Aβ10–20 alone, neurons were induced to express C1q to a similar level as Aβ42 (Figure 5k). The sustained C1q induction by Aβ10–20 was confirmed by RT-PCR for C1q with mRNAs extracted from slices (Figure 6a).

### CD40, IL-8, and MCSF mRNAs are induced by Aβ42 and differentially regulated by Aβ10–20 and APV

It is known that activated microglia cells can produce pro-inflammatory cytokines, chemokines, and nitric oxide, as well as higher expression of co-stimulatory molecules like CD40 and B7 [39]. Many of those proteins have been shown to be upregulated in microglia stimulated by Aβ in cell culture and *in vivo *[40]. Semi-quantitative reverse transcriptase PCR technique was used to determine how certain inducible activation products were modified in slice cultures stimulated with exogenous Aβ42 and in the presence of Aβ10–20 or APV. Rat slices were treated with 30 μM Aβ42 +/-APV or 10 μM Aβ42 +/- 30 μM Aβ10–20 for 3 days before mRNAs were extracted from tissues. LPS, was added at 150 ng/ml for 24 hr, served as positive control, with positive detection for all molecules tested (data not shown).

RT-PCR revealed that mRNAs for CD40 and IL-8 were enhanced in Aβ treated slice cultures relative to the control after 3 days (Figure 6a and 6b). Both Aβ10–20 and APV inhibited Aβ42-triggered upregulation of CD40 (Figure 6a and 6b), consistent with the inhibition of microglial activation by both Aβ10–20 and APV assessed by immunohistochemistry. APV also blocked Aβ42-induced IL-8 expression (Figure 6b), as did Aβ10–20 (data not shown).

Macrophage-colony stimulating factor (MCSF), a proinflammatory mediator for microglial proliferation and activation, has been shown to be expressed by neurons upon Aβ stimulation [41]. The expression of MCSF was induced in slice culture by Aβ treatment by Day 3 (Figure 6a and 6b) and this increase was blocked by the presence of APV (Figure 6b). In contrast, Aβ10–20 did not alter the Aβ42-triggered MCSF induction (Figure 6a), suggesting that MCSF may be required for microglial activation, but alone is not sufficient to induce that activation.

## Discussion

Previously, it has been shown that Aβ is taken up by pyramidal neurons in hippocampal slice culture and that the synthesis of complement protein C1q is induced in neurons [34]. Here we demonstrate that blocking of Aβ42 accumulation in neurons by NMDA receptor antagonist APV and increasing Aβ42 ingestion by integrin antagonist RGD is accompanied by inhibition and elevation in neuronal C1q expression, respectively. However, Aβ10–20, which markedly inhibits Aβ42 accumulation in pyramidal neurons, does not have any inhibitory effect on neuronal C1q expression. Thus, intraneuronal accumulation of Aβ is not necessary for Aβ-mediated induction of neuronal C1q synthesis.

Since Aβ10–20 alone can induce a level of C1q expression in neurons comparable to Aβ42, it is hypothesized that amino acids 10–20 in Aβ peptide contain the sequence that is recognized by at least one Aβ receptor. It was reported by Giulian et al. that the HHQK domain (residues 13–16) in Aβ is critical for Aβ-microglia interaction and activation of microglia, as they demonstrated that small peptides containing HHQK suppress microglial activation and Aβ-induced microglial mediated neurotoxicity [38]. We have previously reported that rat Aβ42, which differs in 3 amino acids from human Aβ42, including 2 in the 10–20 region and 1 in the HHQK domain, was internalized and accumulated in neurons but failed to induce neuronal C1q expression [34]. This is consistent with the hypothesis that a specific Aβ interaction (either neuronal or microglial), presumably via the HHQK region of the Aβ peptide, but not intracellular Aβ accumulation, can lead to neuronal C1q induction in hippocampal neurons.

**Figure 7:**
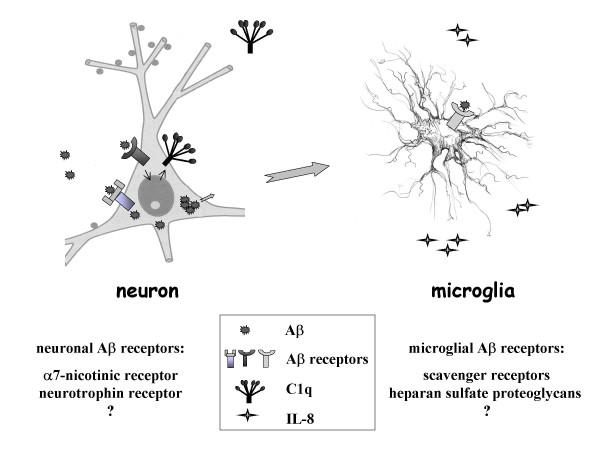
Model of Aβ interaction with neurons and microglia in slice cultures. Exogenous Aβ peptide interacts with neuronal receptors leads to at least two separate consequences, in one of which C1q expression is upregulated in neurons. A second receptor mediates the secretion of certain modulatory molecules, which lead to microglial activation involving the expression of CD45, CR3, CD40, and IL-8. This does not exclude the direct interactions of Aβ with receptor(s) on microglia that may also contribute to microglial activation.

Neurons are the major type of cells that accumulate exogenous Aβ in slice cultures. Microglial activation, as assessed by CD45, OX42, and ED1, was increased with enhanced neuronal Aβ42 uptake and inhibited when Aβ42 uptake was blocked by APV or Aβ10–20 in this slice culture system. These data would be consistent with a model in which neurons, upon internalization of Aβ peptide, secrete molecules to modulate microglial activation [14,41,42] (Figure 7, large arrows). Synthesis and release of those molecules may require the intracellular accumulation of Aβ since blocking intraneuronal Aβ accumulation always blocked microglial activation. The finding that treatment with Aβ10–20 alone did not result in intraneuronal Aβ immunoreactivity or microglial activation, while rat Aβ42, which did accumulate within neurons, induced activation of microglial cells, is consistent with this hypothesis. It should be noted that an absence of Aβ immunoreactivity in Aβ10–20 treated slices does not exclude the possibility that Aβ10–20 was ingested but soon degraded by cells, and thus accumulation of Aβ rather than ingestion alone may be necessary to induce secretion of microglia activating molecules from neurons. Giulian et al. reported that the HHQK region alone was not able to activate microglia [38]. Thus, Aβ10–20 might block microglial activation by competing with Aβ42 for direct microglial binding, as well as by blocking uptake and accumulation of Aβ in neurons.

Activated glial cells, especially microglia, are major players in the neuroinflammation seen in of Alzheimer's disease [43]. Microglial cells can be activated by Aβ and produce proinflammatory cytokines, nitric oxide, superoxide, and other potentially neurotoxic substances *in vitro*, although the state of differentiation/ activation of microglia and the presence of other modulating molecules is known to influence this stimulation [7,9,43]. "Activated" microglia also become more phagocytic and can partially ingest and degrade amyloid deposits in brain. This leads many to hypothesize that there are multiple subsets of "activated" microglia, each primed to function in a specific but distinct way [5,43].

In hippocampal slice cultures, we and others have shown that Aβ42 triggered microglial activation as assessed by immunohistochemical detection of CR3 (OX42), and cathepsin D [34,37]. Several chemokines, including macrophage inflammatory protein-1 (MIP-1α, MIP-1β), monocyte chemotactic protein (MCP-1), and interleukin 8 (IL-8), have been reported to increase in Alzheimer's disease patients or cell cultures treated with Aβ [44,45]. CD40, a co-stimulatory molecule, is also upregulated in Aβ-treated microglia [10]. In this study, similar to reports of cultured microglia, immunoreactivity of CD45 was found increased on microglia in Aβ42 treated slice cultures, and CD40 and IL-8 messenger RNAs were elevated after Aβ42 exposure. As expected, CD40 and IL-8 mRNA induction was blocked whenever immunohistochemistry analysis showed the inhibition of microglial activation. [We did not observe change in MIP-1α, 1β mRNAs in slice culture with Aβ42 treatment, and MCP-1 was too low to be detected with or without Aβ stimulation although it was detectable in LPS treated slices (data not shown).]

The data presented thus far suggest the hypothesis that neurons, upon uptake and accumulation of Aβ, release certain substances that activate microglia. One possible candidate of those neuron-produced substances is MCSF, which has been reported to be induced in neuronal cultures upon Aβ stimulation [41,46], and is known to be able to trigger microglial activation [47]. Indeed, MCSF mRNA was found to increase after 3 days of Aβ treatment (Figure 6a and 6b). The diminished MCSF signal with the addition of APV and coordinate lack of microglial activation is consistent with a proposed role of activating microglia by MCSF produced by stimulated neurons. However, in the presence of Aβ10–20, MCSF induction was unaltered, though microglial activation was inhibited. Thus, MCSF alone does not lead to the upregulation of the above-mentioned microglial activation markers.

In this organotypic slice culture, no significant neuronal damage was observed in 3 day treatment with Aβ at concentrations that have been reported to cause neurotoxicity in cell cultures. One possible explanation is that the peptide has to penetrate the astrocyte layer surrounding the tissue to reach the multiple layers of neurons. Thus, the effective concentration of Aβ on neurons is certainly much lower than the added concentration. Aβ failing to induce neurotoxicity in slices to the same extent as in cell cultures may also indicate the loss of certain protective mechanisms in isolated cells. A distinct advantage of the slice culture model is that the tissue contains all of the cell types present in brain, the cells are all at the same developmental stage, and cells may communicate in similar fashion as *in vivo*.

Our data demonstrating distinct pathways for the induction of neuronal C1q and the activation of microglial by amyloid peptides suggest the involvement of multiple Aβ receptors on multiple cell types in response to Aβ (Figure 7, model) and possibly in Alzheimer's disease progression. This multiple-receptor mechanism is supported by reports suggesting many proteins/complexes can mediate the Aβ interaction with cells [48]. These include, but not limited to, the alpha7nicotinic acetylcholine receptor (alpha7nAChR), the P75 neurotrophin receptor (P75NTR) on neurons, the scavenger receptors and heparan sulfate proteoglycans on microglia, as well as receptor for advanced glycosylation end-products (RAGE) and integrins on both neurons and microglia (Figure 7). Several signaling pathways have been implicated in specific Aβ-receptor interactions [49-51]. However, it is not known which receptors are required for induction of C1q in neurons. In addition, as of yet the function of neuronal C1q has not been determined. Previous reports from our lab have shown that C1q is associated with hippocampal neurons in AD cases but not normal brain [52], and the fact that it is synthesized by the neurons has been documented by others [23,53]. In addition, C1q was prominently expressed in a preclinical case of AD (significant diffuse amyloid deposits, with no plaque associated C1q, and no obvious cognitive disorder) and is expressed in other situations of "stress" or injury in the brain [54-58]. Indeed, overexpression of human cyclooxygenase-2 in mice leads to C1q synthesis in neurons and inhibition of COX-2 activity abrogates C1q induction. These data suggest that in addition to the facilitation of phagocytosis by microglia [59,60] (particularly of dead cells or neuronal blebs), the induction of C1q may be an early response of neurons to injury or regulation of an inflammatory response, consistent with a role in the progression of neurodegeneration in AD. Whether and how the neuronal C1q production affects the survival of neurons is still under investigation. Identifying the receptors responsible for neuronal C1q induction may be informative in understanding the role of C1q in neurons in injury and disease.

## Conclusions

In summary, induction of C1q expression in hippocampal neurons by exogenous Aβ42 is dependent upon specific cellular interactions with Aβ peptide that require HHQK region-containing sequence, but does not require intraneuronal accumulation of Aβ or microglial activation. Thus, induction of neuronal C1q synthesis may be an early response to injury to facilitate clearance of damaged cells, while modulating inflammation and perhaps facilitating repair. Microglial activation in slice culture involves the induction of CD45, CD40, CR3, and IL-8, which correlates with intraneuronal accumulation of Aβ, indicating contribution of factors released by neurons upon Aβ exposure. MCSF may be one of those stimulatory factors, though by itself MCSF cannot fully activate microglia.

Removal of Aβ to prevent deposition and of cellular debris to avoid excitotoxicity would be a beneficial role of microglial activation in AD. However, activated microglia also produce substances that are neurotoxic. Therefore, the goal of modulating the inflammatory response in neurodegenerative diseases like AD is to enhance the phagocytic function of glial cells and inhibit the production of proinflammatory molecules. Being able to distinguish in the slice system C1q expression (which has been shown to facilitate phagocytosis of apoptotic cells in other systems [24]) from microglial activation suggests a plausible approach to reach that goal *in vivo*.

## List of abbreviations

Aβ: amyloid beta; AD: Alzheimer's disease; APV: D-(-)-2-amino-5-phosphonovaleric acid; BSA: bovine serum albumin; GRGDSP (RGD): glycine-arginine-glycine-aspartic acid-serine-proline; HBSS: Hanks' balanced salt solution; HEPES: N-2-hydroxyethylpiperazine-N'-2-ethanesulfonic acid; MCSF: macrophage colony stimulating factor; NMDA: N-methyl-D-aspartic acid; PMSF: phenylmethylsulfonylfluoride; TAE: triethanolamine.

## Competing interests

The author(s) declare that they have no competing interests.

## Authors' contributions

RF cultured and processed the tissue, performed all experiments (immunohistochemistry, ELISA, PCR and others), analyzed the data, and drafted the manuscript. AJT contributed to the design of the study, guided data interpretation and presentation and edited the manuscript.
